# Evaluating the impact of public health initiatives on trends in fecal occult blood test participation in Ontario

**DOI:** 10.1186/1471-2407-14-537

**Published:** 2014-07-25

**Authors:** Gladys N Honein-AbouHaidar, Linda Rabeneck, Lawrence F Paszat, Rinku Sutradhar, Jill Tinmouth, Nancy N Baxter

**Affiliations:** Division of Support, System and Outcomes, University Health Network, Toronto, ON Canada; Dalla Lana School of Public Health, University of Toronto, Toronto, ON Canada; Prevention and Cancer Control, Cancer Care Ontario, Toronto, ON Canada; Department of Medicine, University of Toronto, Toronto, ON Canada; Institute for Health Policy Management and Evaluation, University of Toronto, Toronto, ON Canada; Institute for Clinical Evaluative Sciences, Toronto, ON Canada; Sunnybrook Research Institute, Toronto, ON Canada; ColonCancerCheck Program, Cancer Care Ontario, Toronto, ON Canada; Department of Surgery and Li Ka Shing Knowledge Institute, St. Michael’s Hospital, Toronto, ON Canada

**Keywords:** Public health policy, Colorectal cancer screening, Epidemiologic study

## Abstract

**Background:**

Since the publication of two randomized controlled trials (RCT) in 1996 demonstrating the effectiveness of fecal occult blood test (FOBT) in reducing colorectal cancer (CRC) mortality, several public health initiatives have been introduced in Ontario to promote FOBT participation. We examined the effect of these initiatives on FOBT participation and evaluated temporal trends in participation between 1994 and 2012.

**Method:**

Using administrative databases, we identified 18 annual cohorts of individuals age 50 to 74 years eligible for CRC screening and identified those who received FOBT in each quarter of a year. We used negative binomial segmented regression to examine the effect of initiatives on trends and Joinpoint regression to evaluate temporal trends in FOBT participation.

**Results:**

Quarterly FOBT participation increased from 6.5 per 1000 in quarter 1 to 41.6 per 1000 in quarter 72 (January-March 2012). Segmented regression indicated increases following the publication of the RCTs in 1996 (Δ slope = 6%, 95% CI = 4.3-7.9), the primary care physician financial incentives announcement in 2005 (Δ slope = 2.2%, 95% CI = 0.68-3.7), the launch of the ColonCancerCheck (CCC) Program (Δ intercept = 35.4%, 95% CI = 18.3 -54.9), and the CCC Program 2-year anniversary (Δ slope = 7.2%, 95% CI = 3.9 – 10.5). Joinpoint validated these findings and identified the specific points when changes occurred.

**Conclusion:**

Although observed increases in FOBT participation cannot be definitively attributed to the various initiatives, the results of the two statistical approaches suggest a causal association between the observed increases in FOBT participation and most of these initiatives.

**Electronic supplementary material:**

The online version of this article (doi:10.1186/1471-2407-14-537) contains supplementary material, which is available to authorized users.

## Background

The population health burden of colorectal cancer (CRC) in Canada is substantial [1]. In Ontario, Canada, CRC is the second cause of cancer mortality [1]. Screening for CRC can reduce the burden of this disease. Three landmark randomized controlled trials (RCTs) published between 1993 and 1996 demonstrated that biennial use of the fecal occult blood test (FOBT), coupled with colonoscopy in those who test positive, resulted in a 15% reduction in CRC mortality [2–4]. The publication of these RCTs motivated policy makers to make various efforts to promote FOBT participation in Ontario.

In February 2001, the Canadian Task Force on Preventive Health Care (CTFPHC) published guidelines recommending FOBT as a CRC screening test for average risk individuals aged 50 to 74 years (Level A Recommendation) [5]. The dissemination of these guidelines into clinical practice was passive and without any mechanism to promote adherence.

In July 2005, the Ministry of Health and Long-Term Care (MOHLTC) of Ontario announced new financial incentives for CRC screening targeting primary care physicians (PCPs) in patient enrolment model (PEM) types of practice (50% of Ontario physicians at that time) [6]. Eligible PCPs received end of fiscal year bonuses based on the proportion of enrolled patients who received FOBT prior to March 31^st^ of each year. The bonus amount increases as the proportion of screened patients increases, e.g. if 20% of enrolled patients are screened, the PCP receives $440; if 50% are screened, the PCP receives $2,200. The first bonus submission was on April 1^st^ of 2006 for FOBT screening of enrolled patients from April 1 2005 through March 31 2006 [7].

In April 2008, Cancer Care Ontario, Ontario’s provincial cancer agency responsible for cancer services, and the MOHLTC launched the ColonCancerCheck (CCC) Program, the first province-wide organized CRC screening program in Canada. The CCC Program recommends FOBT every 2 years for average risk individuals age 50 to 74 years and colonoscopy for those who test positive [8]. An intense but temporary public media campaign and a PCP educational program marked the launch of the CCC Program. Starting from fiscal year 2008, PCPs became eligible to receive up to $4,000 if 70% of their enrolled patients were screened [9–12].

April 2010 marked the CCC Program 2- year anniversary. In addition to ongoing PCP screening practices, the CCC Program rolled out recall and reminder letter interventions. Recall letters were sent out to those who were FOBT negative in the first round of screening inviting them to be re-screened. These recall letters were sent in August 2010 for those who completed FOBT in the previous 24–30 months and in December 2010, a reminder letter was sent for those who had not yet undergone FOBT screening [13].

The goal of this population-based time trend study was to examine the effect of the publication of the RCTs and the CTFPHC guidelines, the announcement of PCP financial incentives, the launch of the CCC Program, and the programmatic correspondence following the CCC Program 2-year anniversary on FOBT participation in Ontario and to evaluate temporal trends in FOBT participation between April 1^st^ 1994 and March 31^st^ 2012.

## Methods

The Research Ethics Board of St. Michael’s Hospital in Toronto approved this study.

### Data sources

We used four data holdings including the Registered Persons Database (RPDB), the Ontario Health Insurance Plan (OHIP) database, the Ontario Cancer Registry (OCR), and the Canadian Institute for Health Information Discharge Abstract Database (CIHI-DAD). These data holdings are housed at the Institute for Clinical Evaluative Sciences (ICES) [14]. Each data record collected at ICES comes with personal identifier, usually a health card number. Using a secure ICES algorithm, each health card number is assigned a unique encrypted ICES number (IKN). Once records in a data set have an IKN assigned, the identifying information is stripped off the file and the data become de-identified. Researchers have access to the de-identified data only. The unique IKN is used to link the various data sets.

The RPDB is a roster of all permanent residents and refugees eligible for coverage under the Ontario Health Insurance Plan, which contains demographic information including an individual’s date of birth, sex, date of death (where applicable), and changes in eligibility for health insurance coverage. The OHIP database contains information about all claims for physician and laboratory services provided to Ontario residents since July 1991. The OCR is a registry of all Ontario residents diagnosed with cancer since 1964. The OCR captures over 95% of cancer cases in Ontario [15]. The CIHI-DAD contains information from hospitalization records, abstracted since April 1988.

### Study cohorts

All persons eligible for OHIP aged 50 to 74 years were identified from the RPDB at the beginning of each fiscal year from 1994 to 2012. Using IKN, we linked these cohorts to OCR and CIHI-DAD to exclude individuals diagnosed with CRC or Inflammatory Bowel Disease before April 1st of each year to approximate cohorts of individuals at average risk for CRC. (Additional file 1: Diagnostic and OHIP procedure codes).

We used OHIP database to identify those who received CRC screening tests in each fiscal year and in the previous ten years (Additional file 1). For persons with multiple claims in a fiscal year, we included the first service date for FOBT; for persons with multiple claims in the previous 10 years we included the most recent service date for this time period.

The data were analyzed by quarter of a fiscal year. For each quarter, we included all individuals due for CRC screening in our denominator; individuals who underwent FOBT during the quarter formed our numerator. We applied the following exclusions to approximate a population that was due for CRC screening:

1. At the beginning of each quarter, we excluded those who died in the previous quarter(s) of the same year;
2. At the end of each quarter, we excluded those who were up-to-date with CRC screening as defined as having: FOBT within two years; a flexible sigmoidoscopy or barium enema within five years; or a colonoscopy within ten years.

### Statistical analysis

We used two statistical methods. We used a segmented regression analysis to compare changes in trends in FOBT participation before and after initiatives including: publication of RCTs (1996), publication of the CTFPHC guidelines (2001), announcement of PCP financial incentives (2005), launch of the CCC Program (2008), and the programmatic correspondence following the CCC Program 2-year anniversary (2010). In this analysis, a dummy variable (INT) coded 0 before and 1 after the expected time of each intervention, and an interaction term (INT*Timeafter) were added to the model as suggested by Wagner et al. [16]. The dummy variable (INT) indicates change in intercept, the interaction term indicates change in slope (Detailed procedure of statistical analysis is shown in Additional file 2). A change in slope or intercept was considered statistically significant if the 95% confidence interval did not include zero. Data were analyzed using SAS software 9.3. [17].

Because segmented regression uses pre-defined points, the results may mask the specific date when the actual change in trend occurred [18]. We, therefore, conducted a Joinpoint regression (ver. 4.0) a technique that enables trend modeling without pre-defined points [19, 20]. We fitted the joinpoint regression model as follows: we used FOBT count in each quarter as the numerator, individuals due for CRC screening (denominator) as an “offset term”, and the quarter as the regressor variable. We estimated the quarterly percent change (QPC), i.e. rate of change in slope between joinpoints, the intercept of each joinpoint, and corresponding 95% confidence intervals using the following parameters: 1) Grid Search method; 2) Bayesian Information Criteria model selection method; 3) up to 6 joinpoints for each model; 4) a minimum of 5 quarters between two joinpoints; and 5) Poisson variance [21]. The trend was considered statistically significant if the 95% confidence interval of the QPC did not include zero [18, 20–24].

## Results

### Cohort characteristics

**Figure 1:**
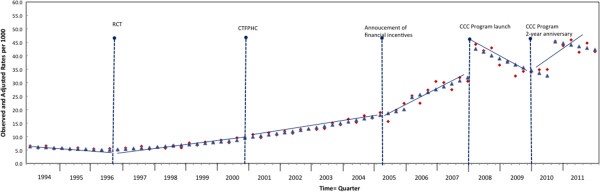
**Observed rates and segmented regression adjusted rates of fecal occult blood test (FOBT) participation per 1000, Ontario, 1994–2012.** Observed rate = (FOBT completed per quarter/ population due for CRC screening per quarter)* 1000. Adjusted rate = (Exp (log rate-offset))*1000. Rates are connected by a binomial regression line. Dashed vertical lines indicate quarter when the following initiatives were enacted: RCT: Publication of the second and third randomized controlled trials in November 1996. CTFPHC: Publication of the Canadian Task Force on Preventive Health Care guidelines for CRC screening in February 2001. Announcement of PCP financial incentives in July 2005. CCC Program launch, April 2008. CCC Program 2-year anniversary, April 2010. The regression model was expressed as: .

From fiscal year 1994 to 2012, there were 72 quarters. In each quarter, we identified 198,000 to 207,000 individuals due for CRC screening. Quarterly FOBT participation increased from 6.5 in quarter 1 to 41.6 per 1000 in quarter 72 with a peak in quarter 69 (April-June, 2011), after the programmatic correspondence of the CCC Program (45.9 per 1000). Figure 1 demonstrates an overall increase in FOBT participation between 1994 and 2012 that was not uniform throughout the time period. Participation slowly increased between 1996 and 2005; more rapid increases occurred after 2005.

### Segmented regression results

We plotted the observed and adjusted quarterly rates of FOBT participation in each quarter (Figure 1). The results of the segmented regression analysis are shown in Table 1.

**Table 1:** **Segmented regression analysis showing changes in intercept and changes in slope on FOBT participation rates following each initiative, 1994-2012**

Initiative (Segment)	Intercept ^*^		Slope ^^^	
	Change in Intercept (Δ)	95% CI	Change in slope (Δ)	95% CI
Baseline (April 94-October 96)			−2.4^¥‡^	(−3.9--0.9)
RCT (October 96-January 01)	−3.2	(−13.1-7.9)	6.1^‡^	(4.3-7.9)
CTFPHC (January 01-July 05)	6.8	(−2.8-17.3)	0.3	(−0.6–1.3)
FI (July 05- April 08)	−1.5	(−11.9–10.1)	2.2^‡^	(0.7– 3.8)
CCC launch (April 08-April 10)	35.4^‡^	(18.3–54.9)	−9.7^‡^	(−12--7.4)
2-year anniversary (April 10 –March 12)	13.5	(−1.6–30.9)	7.2^‡^	(3.9–10.5)

*Difference between pre and post initiative intercepts interpreted as step change and calculated as QPC = (exp β_INTi_ -1 )* 100.

^Difference between pre and post initiative slopes taking into account the trend before the initiative and calculated as QPC = (exp β_INT*TIMEi_ -1 )* 100.

¥Baseline slope.

‡Statistically significant if 95% confidence interval does not cross zero.

RCT: Publication of the second and third randomized controlled trials in November 1996.

CTFPHC: Publication of the Canadian Task Force on Preventive Health Care guidelines for CRC screening in February 2001.

FI: Announcement of PCP financial incentives in July 2005.

CCC launch: ColonCancer Check program (CCC) Program launch, April 2008.

2 - year anniversary: ColonCancerCheck Program 2-year anniversary, April 2010.

There was a statistically significant increase in slope in FOBT participation following the publication of the RCTs in 1996 (change in slope = 6.1%, 95% CI = 4.3-7.9), and the announcement of PCP financial incentives (change in slope = 2.2%, 95% CI = 0.7-3.8). The launch of the CCC Program was associated with increase in intercept (change in intercept = 35.4%, 95% CI = 18.3-54.9) followed by a decrease in slope (change in slope = −9.75%, 95% CI = −12-7.4). An increase in slope was detected following the CCC Program correspondence in 2010 (change in slope: 7.2%, 95% CI = 3.9-10.5). Other changes in intercept and slope were not statistically significant (Table 1).

### Joinpoint results

**Figure 2:**
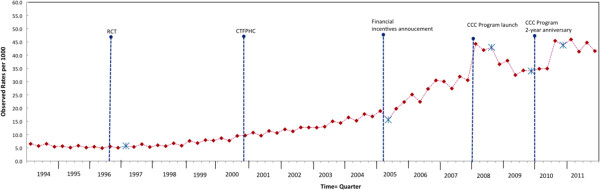
**Observed rates of FOBT participation per 1000 and joinpoint location determined by Joinpoint regression analysis, Ontario, 1994–2012.** Observed rate = (FOBT completed per quarter/population due for CRC screening per quarter)* 1000. * joinpoint location. Dashed vertical lines indicate quarter when the following initiatives were enacted: RCT: Publication of the second and third randomized controlled trials in November 1996. CTFPHC: Publication of the Canadian Task Force on Preventive Health Care guidelines for CRC screening in February 2001. Announcement of PCP financial incentives in July 2005. CCC Program launch, April 2008. CCC Program 2-year anniversary, April 2010.

We plotted the observed rates of FOBT participation per quarter and the Joinpoint location in Figure 2. The results of the Joinpoint regression analysis are shown in Table 2.

**Table 2:** **Joinpoint regression analysis for FOBT participation in Ontario, 1994–2012 showing actual intercept at each joinpoint and actual slope between joinpoints**

Identified segment	Join-point	Intercept		Slope	
		Intercept ^£^	95% CI	QPC ^^^	95% CI
April 1994-April 1997		0.3	(0.15-0.47)	−1.7^¥^	(−3.9–0.5)
April 1997-July 2005	Q 13	0.15^‡^	(0.02-0.28)	3.8^‡^	(3.4–4.2)
July 2005-October 2009	Q 46	0.03	(−0.47-0.53)	7.4^‡^	(6.4–8.5)
October 2009-January 2010	Q 59	62.1^‡^	(59–64.9)	−5.5^‡^	(−9.9--0.9)
January 2010-January 2011	Q 64	0.01	(−4.5-4.5)	8.2^‡^	(0.9-16)
January 2011-March 2012	Q 68	6^‡^	(3.1-8.9)	−1.4	(−5.5–2.9)

^£^Intercept at each joinpoint calculated as (exp β _INTERCEPT i_).

^Quarterly percent change (slope) between joinpoints calculated as (exp β _SLOPEi_ -1)* 100.

¥Baseline trend.

‡Statistically significant if 95% confidence interval does not cross zero.

Joinpoint regression analysis identified five joinpoints and six distinct segments. The change in slopes between Joinpoints and those from the segmented regression analysis converged. Joinpoint regression identified the specific point in time when change occurred, the slope between joinpoints, and the intercept at each joinpoint. An increase in slope started two quarters following RCT publication in 1996 (QPC = 3.8%, 95% CI = 3.4-4.2), followed by another increase in slope starting from the quarter PCP financial incentives were announced (QPC = 7.4%, 95% CI = 6.4-8.5). There was an immediate increase in intercept following the CCC Program launch (Intercept = 62.1, 95% CI: 59-64.9), a decrease in slope three quarters after the launch (QPC = −5.5%, 95% CI = −9.9-0.9), and an increase in slope one quarter before the CCC Program 2-year anniversary (QPC = 8.2%, 95% CI = 0.9-16) (Table 2).

## Discussion

Since 1994, FOBT participation has increased substantially in Ontario. We observed an overall increase in quarterly participation from 6.5 per 1000 in April 1994 to 41.6 per 1000 in March, 2012. Participation slowly increased between 1994 and 2005 followed by a more rapid increase between 2005 and 2012. Although we cannot definitively attribute the observed increases in FOBT participation to the initiatives made to promote participation, the convergence of the two statistical approaches suggest a causal association between the observed increases in FOBT participation and the publication of the RCTs, introduction of PCP financial incentives and CCC Program launch and programmatic correspondence, but not publication of the CTFPHC guideline.

We previously reported the results of a segmented regression to investigate the effect of the launch of the CCC Program on FOBT participation in Ontario over a 6 year time period (2005 to 2011) [25]. Our current study improves upon this analysis by evaluating 18 years of data allowing examination of initiatives before CCC Program launch, enabling the evaluation of CCR program in context of previous trends in FOBT uptake, and evaluation of the programmatic correspondence of the 2-year anniversary of the CCC Program. Further, this study uses two different statistical approaches, each with specific advantages. Segmented regression analysis allowed us to estimate the changes in intercepts and slopes following each intervention accounting for baselines trends, a robust method for measuring the effect of an intervention when randomization or identification of a control group are impractical [16, 26–28]. Joinpoint analysis enabled identification of specific points in time when changes occurred, and provided estimates of the actual intercept and slope for each segment.

Previously, we reported a significant increase in FOBT participation (change in intercept) immediately following the launch of the CCC Program; we attributed the increase to the public media campaign [25]. This increase was followed by a downtrend at the end of the screening period, a concern for policy makers (Dr. Linda Rabeneck, personal communication, January 2009). In the current study, we found that this downtrend was reversible and was observed again after the CCC Program 2-year anniversary, i.e. a peak after the programmatic correspondence followed by a drop at the end of the study period. Fluctuation in trends following the introduction of public policies are reported in the literature [29]. In this instance, however, a periodic trend in FOBT participation with a peak every 2 years has likely been introduced, in keeping with the date of program launch and program recommendation of biennial FOBT screening. Future studies need to examine if this biennial periodicity will persist and the impact on endoscopic and surgical resources.

In 1996, results of RCTs demonstrated that screening with FOBT reduces CRC mortality and in 2001 the CTFPHC strongly endorsed CRC screening with FOBT. Given this evidence, why increases in FOBT participation before 2005 were modest? Integration of evidence into clinical practice has always been challenging [30–32]. Davis et al. indicated that in order for guidelines to be translated into practice, there must be intervention strategies to reinforce their adoption such as reminder systems and academic detailing [31]. Passive strategies, including mailing or publication of guidelines, have little impact on adoption [31]. Because there was no mechanism to actively promote the CTFPHC guidelines, the modest increase in the use of FOBT after their publication is not surprising.

We demonstrated a marked change in participation following the introduction of financial incentives and the programmatic correspondence after the CCC Program 2-year anniversary, indicating these initiatives were likely the reasons for the rapid increase in participation after 2005. In terms of financial incentives, studies show mixed effects on performance varying between no effect at all [33, 34] and improved performance [35–38]. Certain factors have proven to be effective in improving performance. Custers et al. indicate that financial incentives that take into account the size of the bonus, and baseline performance often succeed in improving performance [39]. In this study, two factors may explain the improved performance. First, the size of the reward may have motivated some physicians to change their screening routines [34, 40]. Second, when baseline performance is relatively modest, the introduction of bonuses is more likely to have an impact [41]. Our findings that participation increased following the programmatic correspondence are consistent with those from previous studies that suggested that reminder letters were associated with increased screening participation [42–46].

Our study has limitations. In observational studies, it is difficult to infer a causal association between an intervention and observed trends [47]. We are examining changes in FOBT participation occurring in a complex health system, and factors other than those evaluated in this study may have contributed to changes in trend. However, segmented regression analysis controls for secular trends, i.e. reasons other than the effect of initiatives, by introducing a term in the model to test the effect of the intervention over and above the secular trend [16, 48].

## Conclusion

FOBT participation in Ontario slowly increased between 1994 and 2005 followed by a more rapid increase between 2005 and 2012. The results of the two statistical methods suggest a causal association between those increases and publication of the RCTs, introduction of PCP financial incentives and CCC Program launch and programmatic correspondence, but not the CTFPHC guideline publication. We particularly observed a marked increase after the introduction of the CCC Program in 2008. Although this increase cannot be solely attributed to the CCC Program, evidence from the literature suggests that organized screening programs are effective in increasing participation. Furthermore, we noted a marked increase following the programmatic correspondence after the CCC Program 2-year anniversary. With the information available, it is reasonable to conclude that the marked increase in participation since 2008 might well reflect the impact of the CCC Program on FOBT participation.

## Electronic supplementary material

Additional file 1: **Diagnostic and Ontario Health Insurance (OHIP) procedure codes.** ^*^International Classification of Diseases, 9^th^ and 10^th^ revisions, Clinical Modification. (DOC 39 KB)

Additional file 2: **Detailed procedure of statistical analysis.** (DOC 64 KB)

## Abbreviations

CRC: Colorectal cancer
RCTs: Randomized controlled trials
FOBT: Fecal occult blood test
CTFPHC: Canadian Task Force on Preventive Health Care
MOHLTC: Ministry of Health and Long-Term Care
PCPs: Primary care physicians
PEM: Patient enrolment model
CCC: ColonCancerCheck
RPDB: Registered persons database
OHIP: Ontario Health Insurance Plan
OCR: Ontario Cancer Registry
CIHI-DAD: Canadian Institute for Health Information Discharge Abstract Database
ICES: Institute for Clinical Evaluative Sciences
IKN: Encrypted ICES number.

## References

[1] Canadian Cancer Society’s Advisory Committee on Cancer Statistics (2013). Canadian Cancer Statistics 2013.

[2] Mandel JS, Bond JH, Church TR, Snover DC, Bradley GM, Schuman LM, Ederer F (1993). Reducing mortality from colorectal cancer by screening for fecal occult blood. Minnesota Colon Cancer Control Study. N Engl J Med.

[3] Kronborg O, Fenger C, Olsen J, Jorgensen OD, Sondergaard O (1996). Randomised study of screening for colorectal cancer with faecal- occult-blood test. Lancet.

[4] Hardcastle JD, Chamberlain JO, Robinson MH, Moss SM, Amar SS, Balfour TW, James PD, Mangham CM (1996). Randomised controlled trial of faecal-occult- blood screening for colorectal cancer. Lancet.

[5] CTFPH (2001). Canadian task force on preventive health care.

[6] Kantarevic J, Kralj B, Weinkauf D (2011). Enhanced fee-for-service model and physician productivity: evidence from Family Health Groups in Ontario. J Health Econ.

[7] Li J, Hurley J, Decicca P, Buckley G (2013). Physician Response to Pay- for-Performance: Evidence from a Natural Experiment. Health Econ.

[8] *Colon Cancer Check 2008 Program Report*. Ontario: Toronto Cancer Care; 2010. [September 11, 2013]; Available from: http://www.cancercare.on.ca

[9] *Ministry of Health and Long Term Care Teams Preventive Care Bonus-Tracking and Exclusion Codes*. 2006. [cited February 2013]; Available from: http://www.anl.com/pages/mohguide.htm

[10] *Colon Cancer Check Fecal Occult Blood Testing (FOBT) (Bulletin 4471). [Bulletin]*. 2008. [February 2013]; Available from: http://www.health.gov.on.ca/en/pro/programs/coloncancercheck/role.aspx

[11] *Colon Cancer Check Fecal Occult Blood Testing (FOBT), (Bulletin 4482) [Bulletin]*. 2008. [February 2013]; Available from: http://www.health.gov.on.ca/en/pro/programs/coloncancercheck/role.aspx

[12] *Partnering to Successfully Launch Ontario’s Colorectal Screening Program*. Cancer Care Ontario: Colon Cancer Check regional primary care engagement report; 2009.

[13] *ColonCancerCheck*. [Accessed September 2011 Accessed September 2011]; Available at http://health.gov.on.ca/en/ms/coloncancercheck/

[14] *Institute for Clinical Evaluative Sciences*. Toronto; 2013. Available from: http://www.ices.on.ca/

[15] Robles SC, Marrett LD, Clarke EA, Risch HA (1988). An application of capture-recapture methods to the estimation of completeness of cancer registration. J Clin Epidemiol.

[16] Wagner AK, Soumerai SB, Zhang F, Ross-Degnan D (2002). Segmented regression analysis of interrupted time series studies in medication use research. J Clin Pharm Ther.

[17] *The Data Analysis for This Paper was Generated Using SAS/STAT Software. Version 9.3*. Cary, NC, USA: SAS Institute Inc; 2013.

[18] Gagne M, Robitaille Y, Hamel D, St-Laurent D (2010). Firearms regulation and declining rates of male suicide in Quebec. Inj Prev.

[19] Kim HJ, Fay MP, Feuer EJ, Midthune DN (2000). Permutation tests for joinpoint regression with applications to cancer rates. Stat Med.

[20] *National. Cancer Institute. Joinpoint Regression Program, Version 4.0 [Internet]*. Bethesda, MD: National Cancer Institute; 2013. [cited 2013 June 10]; Available from: http://surveillance.cancer.gov/joinpoint/

[21] Zhichang JZ, Zhenguo Q, Hatcher J: *Joinpoint Trend Analysis of Cancer Incidence and Mortality Using Alberta Data Alberta Canadian Partnership Against Cancer*. *Available from:* http://www.cancerview.ca/idc/groups/public/documents/webcontent/csen_cproj_fy0910q3_joinpoint.pdf

[22] Lee YC, Huang YT, Tsai YW, Huang SM, Kuo KN, McKee M, Nolte E (2010). The impact of universal National Health Insurance on population health: the experience of Taiwan. BMC Health Serv Res.

[23] Edwards BK, Ward E, Kohler BA, Eheman C, Zauber AG, Anderson RN, Jemal A, Schymura MJ, Lansdorp-Vogelaar I, Seeff LC, van Ballegooijen M, Goede SL, Ries LA (2010). Annual report to the nation on the status of cancer, 1975–2006, featuring colorectal cancer trends and impact of interventions (risk factors, screening, and treatment) to reduce future rates. Cancer.

[24] Marrett LD (2009). User Documentation for Surveillance Analytic Software: JoinPoint: Cancer Care Ontario.

[25] Honein-Abouhaidar GN, Baxter NN, Moineddin R, Urbach DR, Rabeneck L, Bierman AS (2013). Trends and inequities in colorectal cancer screening participation in Ontario, Canada, 2005–2011. Cancer Epidemiol.

[26] Lagarde M (2012). How to do (or not to do) … Assessing the impact of a policy change with routine longitudinal data. Health Policy Plan.

[27] Shardell M, Harris AD, El-Kamary SS, Furuno JP, Miller RR, Perencevich EN (2007). Statistical analysis and application of quasi experiments to antimicrobial resistance intervention studies. Clin Infect Dis.

[28] Zarychanski R, Dennis J, Singh H (2013). Challenges of population-based colorectal cancer screening and the importance of time-trend analysis when evaluating system change. Cancer Epidemiol.

[29] Wilson N, Thomson G, Grigg M, Afzal R (2005). New smoke-free environments legislation stimulates calls to a national Quitline. Tob Control.

[30] Anderson LM, May DS (1995). Has the use of cervical, breast, and colorectal cancer screening increased in the United States?. Am J Public Health.

[31] Davis DA, Taylor-Vaisey A (1997). Translating guidelines into practice. A systematic review of theoretic concepts, practical experience and research evidence in the adoption of clinical practice guidelines. CMAJ.

[32] Grimshaw JM, Russell IT (1993). Effect of clinical guidelines on medical practice: a systematic review of rigorous evaluations. Lancet.

[33] Hillman AL, Ripley K, Goldfarb N, Nuamah I, Weiner J, Lusk E (1998). Physician financial incentives and feedback: failure to increase cancer screening in Medicaid managed care. Am J Public Health.

[34] Grady KE, Lemkau JP, Lee NR, Caddell C (1997). Enhancing mammography referral in primary care. Prev Med.

[35] Rosenthal MB, Frank RG, Li Z, Epstein AM (2005). Early experience with pay-for-performance: from concept to practice. JAMA.

[36] Doran T, Fullwood C, Gravelle H, Reeves D, Kontopantelis E, Hiroeh U, Roland M (2006). Pay-for-performance programs in family practices in the United Kingdom. N Engl J Med.

[37] Grossbart SR (2006). What’s the return? Assessing the effect of “pay- for-performance” initiatives on the quality of care delivery. Med Care Res Rev.

[38] Kouides RW, Bennett NM, Lewis B, Cappuccio JD, Barker WH, LaForce FM (1998). Performance-based physician reimbursement and influenza immunization rates in the elderly. Am J Prev Med.

[39] Custers T, Hurley J, Klazinga NS, Brown AD (2008). Selecting effective incentive structures in health care: A decision framework to support health care purchasers in finding the right incentives to drive performance. BMC Health Serv Res.

[40] Town R, Kane R, Johnson P, Butler M (2005). Economic incentives and physicians’ delivery of preventive care: a systematic review. Am J Prev Med.

[41] Sabatino SA, Habarta N, Baron RC, Coates RJ, Rimer BK, Kerner J, Coughlin SS, Kalra GP, Chattopadhyay S (2008). Interventions to increase recommendation and delivery of screening for breast, cervical, and colorectal cancers by healthcare providers systematic reviews of provider assessment and feedback and provider incentives. Am J Prev Med.

[42] Lee JK, Reis V, Liu S, Conn L, Groessl EJ, Ganiats TG, Ho SB (2009). Improving fecal occult blood testing compliance using a mailed educational reminder. J Gen Intern Med.

[43] Eaker S, Adami HO, Granath F, Wilander E, Sparen P (2004). A large population-based randomized controlled trial to increase attendance at screening for cervical cancer. Cancer Epidemiol Biomarkers Prev.

[44] Morrell S, Taylor R, Zeckendorf S, Niciak A, Wain G, Ross J (2005). How much does a reminder letter increase cervical screening among under-screened women in NSW?. Aust N Z J Public Health.

[45] King ES, Rimer BK, Seay J, Balshem A, Engstrom PF (1994). Promoting mammography use through progressive interventions: is it effective?. Am J Public Health.

[46] Page A, Morrell S, Chiu C, Taylor R, Tewson R (2006). Recruitment to mammography screening: a randomised trial and meta-analysis of invitation letters and telephone calls. Aust N Z J Public Health.

[47] Shadish WR, Cook TD, Campbell DT (2002). Experimental and Quasi- Experimental Designs for Generalized Causal Inference.

[48] Ramsay CR, Matowe L, Grilli R, Grimshaw JM, Thomas RE (2003). Interrupted time series designs in health technology assessment: lessons from two systematic reviews of behavior change strategies. Int J Technol Assess Health Care.

[CR49] The pre-publication history for this paper can be accessed here:http://www.biomedcentral.com/1471-2407/14/537/prepub

