# Supplementary material for: Evaluating the impact of public health initiatives on trends in fecal occult blood test participation in Ontario
Source: BMC Cancer. 2014 Jul 25;14:537. doi: 10.1186/1471-2407-14-537 (PMC4132913; doi:10.1186/1471-2407-14-537)
Supplement: Supplementary file 1 — Additional file 1: Diagnostic and Ontario Health Insurance (OHIP) procedure codes. *International Classification of Diseases, 9th and 10th revisions, Clinical Modification. (DOC 39 KB) [file 12885_2014_4755_MOESM1_ESM.doc]

Additional file 1: Diagnostic and Ontario Health Insurance (OHIP) procedure codes.

| Diagnostic codes  ICD-9-CM^*^  CRC  Ulcerative colitis  Crohn’s disease | 153.0-153.4, 153.6-154.1  556, 556.0-556.9  555, 555.0-555.9 |
| --- | --- |
| ICD-10-CM^*^  CRC  Ulcerative colitis  Crohn’s disease | C18-C21, C180, C182-C184, C186-C189  K500, K501  K508-K515 |
| OHIP Procedures codes  FOBT- CCC kits  FOBT- other kits  Single contrast barium enema  Double contrast barium enema  Large bowel endoscopy  Flexible sigmoidoscopy using 60 cm scope  Flexible sigmoidoscopy to descending colon  Colonoscopy to splenic flexure  Colonoscopy to hepatic ﬂexure  Colonoscopy to cecum  Colonoscopy into terminal ileum | L179, Q152  L181, G004  X112  X113  Z580  Z555  Z555 plus E740  Z555 plus E740 plus E741  Z555 plus E740 plus E741 plus E747  Z555 plus E740 plus E741 plus E747 plus E705 |
