# Supplementary material for: Evaluating the impact of public health initiatives on trends in fecal occult blood test participation in Ontario
Source: BMC Cancer. 2014 Jul 25;14:537. doi: 10.1186/1471-2407-14-537 (PMC4132913; doi:10.1186/1471-2407-14-537)
Supplement: Supplementary file 2 — Additional file 2: Detailed procedure of statistical analysis. (DOC 64 KB) [file 12885_2014_4755_MOESM2_ESM.doc]

***Additional file 2- Detailed procedure of statistical analysis***

We conducted two statistical analyses to examine the effect of initiatives on FOBT participation. We used a segmented regression analysis to compare changes in trends in FOBT participation before and after initiatives including: the publication of the second and third RCTs in 1996, the publication of the CTFPHC guidelines in 2001, the announcement of PCP financial incentives in 2005, the launch of the CCC Program in 2008 on FOBT participation, and the second CCC Program anniversary in 2010. In this analysis, a dummy variable (INT) coded 0 before and 1 after the expected time of each intervention, and an interaction term (INT*Timeafter) were added to the model as suggested by Wagner et al.(1) . The dummy variable (INT) indicates change in intercept, i.e. an immediate jump or drop in the outcome after the intervention, and the interaction term indicates change in slope, i.e. a statistically significant increase or decrease in the slope of the segment after the intervention as compared with the segment preceding the intervention.

In this study, the number of FOBTs received in each quarter was our outcome variable and the natural logarithm of the number of individuals due for CRC screening in each quarter (denominator) was the “offset term” (2). The negative binomial distribution was used for evidence of overdispersion (3).

We calculated the change in intercept as (exp β_j_ INT -1)* 100; and the change in slope as (exp β_j_ INT*TIME -1)* 100. The 95% confidence interval of each change was calculated using the Wald method and the adjusted rates for each data point were calculated as suggested by the SAS support guide (4) . We performed a Durbin–Watson test and found no evidence of first-order auto-correlation (5). A change in slope or intercept was considered statistically significant if the 95% confidence interval did not include zero. Data were analyzed using SAS software 9.3. (SAS Institute, Cary, North Carolina) (6).

Because segmented regression uses pre-defined points, the results may mask the specific point in time when the actual change in trend occurred (7). We, therefore, conducted a Joinpoint regression (ver. 4.0; http://surveillance.cancer.gov/joinpoint) a technique that enables trend modeling without pre-defined points (8, 9).

For the Joinpoint regression, we set the parameters for fitting the model as follows: we used the FOBT count in each quarter as the numerator, individuals due for CRC screening (denominator) as an “offset term”, and the quarter as the regressor variable. We estimated the quarterly percent change (QPC), i.e. the rate of change in slope between joinpoints, the intercept of each joinpoint, and their corresponding 95% confidence intervals using the following parameters: 1) Grid Search method ; 2) Bayesian Information Criteria model selection method (i.e. The optimal model is the one with the minimum Sum of Squared Error Estimate (SSE)); 3) up to 6 joinpoints for each model; 4) a minimum of 5 quarters between two joinpoints; and 5) Poisson variance (10). In addition, we conducted a series of tests to determine whether our data were auto-correlated and whether the residuals of the observed outcomes follow a linear or log-linear model (8). Since there was no change in the parameter estimates between the auto-correlated and uncorrelated model, we chose the uncorrelated model in order to maintain the power of the test and we selected the log-linear model since it had a lower residual error. The trend was considered statistically significant if the 95% Confidence interval of the QPC did not include zero (7, 9-13).
